# From AI use to critical thinking among medical students: a moderated mediation perspective on cognitive load and self-regulated learning

**DOI:** 10.3389/fpsyg.2026.1883053

**Published:** 2026-08-13

**Authors:** Arooj Arshad, Ayoob Lone, Loveena Arickswamy, Komal Hassan, Abdullah Abdulaziz Alnaim, Mohammed Farhan AlFarhan

**Affiliations:** 1 Riphah Institute of Clinical and Professional Psychology, Riphah International University, Lahore, Pakistan; 2 Department of Clinical Neurosciences, College of Medicine, King Faisal University, Al Ahsa, Saudi Arabia; 3 Amity University, Dubai, United Arab Emirates; 4 Department of Family and Community Medicine, College of Medicine, King Faisal University, Al Ahsa, Saudi Arabia; 5 Orthopedic Division, Department of Surgery, College of Medicine, King Faisal University, Al Ahsa, Saudi Arabia

**Keywords:** AI-based educational technologies, cognitive load, critical thinking, medical students, self-regulated learning

## Abstract

**Background:**

The increasing integration of artificial intelligence (AI) in medical education has raised important questions regarding its impact on higher-order cognitive processes, particularly critical thinking.

**Aim and Objectives:**

This study examines the interplay of cognitive load and self-regulated learning in explaining how AI-based educational technology influences critical thinking among medical university students.

**Methods:**

Using a cross-sectional research design, data were collected from 480 undergraduate medical students with prior experience using AI tools. Data were collected using standardized measures assessing AI usage, cognitive load, self-regulated learning, and critical thinking. Hayes' PROCESS Model 14 was employed to test mediation and moderated mediation effects.

**Results:**

The findings revealed that AI-based technology use was positively associated with critical thinking and self-regulated learning, while cognitive load showed a negative association with both variables. Mediation analysis indicated that cognitive load partially mediated the relationship between AI use and critical thinking. Furthermore, the moderated mediation analysis demonstrated that self- regulated learning significantly moderated the indirect effect, such that the negative impact of cognitive load on critical thinking weakened at higher levels of self-regulation.

**Conclusion:**

These findings highlight that the effectiveness of AI in enhancing critical thinking depends not only on its cognitive support but also on learners' ability to regulate their engagement. The study provides theoretical and practical implications for integrating AI in educational contexts.

## 1 Introduction

The rapid advancement of artificial intelligence (AI) technologies has significantly transformed higher education (Chan and Colloton, 2024). Generative AI tools are increasingly integrated into academic environments, enabling students to access information, generate ideas, summarize materials, and assist with writing. Platforms such as ChatGPT, Claude, and Gemini have gained popularity due to their accessibility and ability to produce human-like responses (Lindell and Stöhr, 2025). These technologies are reshaping how students interact with knowledge and approach learning. While AI tools offer opportunities to enhance efficiency and personalized support, their use raises questions regarding their impact on cognitive development and higher-order thinking skills (Kasneci et al., 2023).

Among the most significant concerns is the effect of AI on critical thinking skills, which are considered fundamental outcomes of higher education (Chan, 2023). Critical thinking involves the ability to evaluate evidence, analyze arguments, identify assumptions, and make rational judgments based on logic and experience (Facione, 1990). While AI can aid in generating ideas and allow users to gain exposure to different viewpoints, AI can also limit the amount of mental processing necessary to critically think through problems/analyze information, therefore increasing the possibility that students will have shallow learning experiences. With an increase in literature focused on AI's impact on education, the majority of the studies are centered around efficiency of learning, academic performance, or student engagement (Wang and Liu, 2025; Wang et al., 2026) rather than understanding the cognitive processes involved in higher-level thinking.

Two important theoretical perspectives that may explain the educational impact of AI are Cognitive Load Theory (CLT) and Self-Regulated Learning (SRL). CLT proposes that learning effectiveness depends on the management of limited working memory resources (Paas and van Merriënboer, 2020). In contrast, SRL focuses on students' capacity to plan, monitor, and control their own learning processes (Zimmerman, 2000; Pintrich, 2004; Tinajero et al., 2024). Both of these processes will ultimately influence whether or not a student's experience with an AI tool enhances or hinders the opportunity to engage in meaningful learning. AI tools can potentially decrease extraneous cognitive load through organizing and summarizing information; however, they can also create additional cognitive burden through the presentation of vast amounts of content that need to be evaluated and validated. Ultimately, the success of AI tools is dependent upon students being able to strategically utilize the resources available (Farinosi and Melchior, 2025). As a result, students who have developed a high level of self-regulated learning abilities are much better equipped to judge, synthesize, and apply AI generated output as a resource to help with cognition rather than a substitute for independent thinking.

Growing concerns regarding the increasing use of AI in higher education have also focused on issues related to academic integrity, reduced cognitive engagement, and dependency on automated systems (Graham et al., 2025). Because generative AI systems can produce highly sophisticated and human-like responses, students may become increasingly dependent on technology for completing learning tasks, potentially limiting opportunities for independent analysis and reflective thinking (Al-Motrif, 2025). Higher education institutions, however, have historically been slow to adapt to new technologies, which creates challenges for policy development, pedagogy, and assessment processes in keeping up with the evolving technology landscape (Aler Tubella et al., 2024). As such, universities are starting to develop guidance documents for promoting ethical AI use and maintaining academic integrity (Karataş et al., 2025), suggesting that there needs to be an overhaul in curriculum design and assessment practice.

Emerging research has demonstrated that the educational effects of AI depend on how AI is implemented in the classroom (Sandu et al., 2025). AI does not need to be limited to providing students with answers, but rather could be used to provide cognitive support during the learning process through encouraging reflection. Therefore, understanding the interplay between how educators utilize AI, their cognitive processes, and the degree to which they self-regulate is imperative in order to determine the extent to which AI impacts higher-order learning outcomes (Lindell and Stöhr, 2025).

Given the potential risks associated with the use of artificial intelligence in educational settings, it is also important to assess whether or not AI supports higher order cognitive processing, specifically critical thinking. Critical thinking is broadly regarded as an overarching goal of post-secondary education and is defined as the capacity to critically think about arguments, assess evidence, identify assumptions, and draw rational conclusions (Wu et al., 2025). Contemporary knowledge societies are defined by rapid information creation and instant access to vast amounts of data. As a result, it is imperative that people develop these skills to navigate their increasingly complex educational and professional lives (Dessingué and Wagner, 2024). Generative AIs offer possibilities for developing critical thinking as they can produce written explanations, summaries and even assist with generating ideas while also providing an opportunity to expose students to multiple viewpoints through inquiry-based learning (Toci et al., 2025; Adnans et al., 2025).

However, there are still some concerns about how easy access to all kinds of AI will affect the amount of mental work that students require for reasoning independently. Many believe that if a student has to get an answer from artificial intelligence, then they will spend much less time engaged with learning material (Kwan and Hung, 2025). The process of “cognitive off-loading” is when people transfer their own mental processes and assign them to technology systems. Cognitive off-loading can create questions concerning the degree to which AI-supported learning environments encourage deep learning or simply support shallow knowledge consumption. Empirical research on AI and critical thinking remains limited, as most studies have focused on efficiency and engagement rather than higher-order cognitive outcomes (Adnans et al., 2025). This highlights the need to examine cognitive mechanisms such as processing demands and self-regulation.

Cognitive Load Theory explains how we learn in complex learning situations by explaining the limitations of our working memory and the importance of managing cognitive loads (Paas and van Merriënboer, 2020). Cognitive load consists of intrinsic, extraneous, and germane components, where intrinsic load reflects task complexity, extraneous load relates to the presentation of information, and germane load involves schema construction (Sweller, 2010; Seufert et al., 2024). Educational technology using artificial intelligence like ChatGPT, presents an additional dynamic to consider when thinking about how learners will be able to organize information and provide scaffolding for students while reducing their cognitive demands. However, at the same time, AI-based educational technologies could potentially add to the learner's cognitive load because they generate large amounts of information that the learner must process and then develop into meaningful knowledge. Therefore, whether or not an AI-based technology is effective depends on how well the learner is able to manage its effects on their cognition (Palomino-Flores et al., 2026).

Self-regulated learning represents another important factor influencing how students engage with AI technologies. Self-regulated learning is particularly critical in digital learning environments where students must independently navigate information sources and learning activities (Ayaz et al., 2026). Previous research has consistently shown that students with stronger self-regulation skills demonstrate greater academic achievement and deeper learning strategies (Ayaz et al., 2026; Kay, 2023).

Generative AI has added new elements to SRL in which learners now have to make an evaluation about whether or not the generated content is credible, when they can trust the AI for support, and how they will incorporate this type of information into their own learning process (Zhou et al., 2024). While research indicates that the degree of a student's reliance upon AI-assisted learning will be highly dependent on their self-regulated learning capacity. It also illustrates that those who are strong in SRL utilize AI as a tool, while students with weak SRL will over-rely upon AI for support (Weigelt et al., 2026).

Building on this perspective, self-regulated learning is expected to influence how learners manage cognitive load while interacting with AI-supported learning environments. Learners who have strong levels of self-regulated learning are well-equipped to control cognitive demands using planning, monitoring and adaptive strategies; thus, they will remain engaged with complex tasks (Dong et al., 2025 and Kay, 2023). On the other hand, learners who have weak levels of self-regulated learning will likely experience cognitive overload or will use AI outputs without criticism (Zhou et al., 2024). In this manner, self-regulated learning can function as a mediator and moderator of the relationship between cognitive load and critical thinking. Cognitive load and self-regulation represent a holistic, integrative model of how AI-based educational technologies impact critical thinking.

### 1.1 Hypotheses development

Building on Cognitive Load Theory and Self-Regulated Learning theory, the present study proposes a moderated mediation model to examine the relationships among AI-based educational technology use, cognitive load, self-regulated learning, and critical thinking. Cognitive Load Theory emphasizes the limited capacity of working memory and the importance of managing cognitive demands during learning (Paas and van Merriënboer, 2020), whereas self-regulated learning theory emphasizes learners' active regulation of cognition, motivation, and behavior through processes such as planning, monitoring, and self-evaluation (Zimmerman, 2000; Pintrich, 2004). Consistent with the aims of the study, the proposed hypotheses focus on the direct relationship between AI-based educational technology use and critical thinking, the relationship between cognitive load and critical thinking, the mediating role of cognitive load, and the moderating role of self-regulated learning in the indirect relationship between AI-based educational technology use and critical thinking.

AI-based educational technology may influence students' critical thinking by providing access to information, explanations, alternative perspectives, and interactive learning support. When students engage actively with AI-generated information, they may compare different viewpoints, evaluate evidence, and critically assess the information provided. Such processes are consistent with the core components of critical thinking, including analysis, evaluation, and inference (Facione, 1990). Recent scholarship on generative AI in higher education similarly suggests that the educational value of AI depends substantially on how learners engage with and evaluate AI-generated content rather than simply accepting it as an unquestioned source of information (Kasneci et al., 2023). Therefore, the use of AI-based educational technology is expected to be positively associated with critical thinking.

*H1: AI-based educational technology use is positively associated with critical thinking*.

Cognitive Load Theory suggests that excessive cognitive demands may consume the limited working-memory resources required for analysis, evaluation, reasoning, and decision-making (de Jong, 2010; Sweller, 2010). When cognitive demands exceed available cognitive capacity, learners may have fewer resources available for higher-order thinking and may be less able to critically evaluate information (Paas and van Merriënboer, 2020). Accordingly, higher cognitive load is expected to be associated with lower levels of critical thinking.

*H2: Cognitive load is negatively associated with critical thinking*.

The preceding arguments suggest that cognitive load may explain how AI-based educational technology use is associated with critical thinking. AI-based educational technologies may alter the amount and nature of cognitive processing required during learning. AI tools may reduce extraneous processing by organizing, summarizing, or explaining information; however, learners may also need to evaluate the accuracy, relevance, and credibility of AI-generated information. These processes may change the cognitive demands experienced during learning and subsequently influence students' capacity to engage in higher-order thinking. Thus, cognitive load is proposed as a mediator of the relationship between AI-based educational technology use and critical thinking. This proposition is consistent with CLT, which conceptualizes learning outcomes as partly dependent on the relationship between task demands, available cognitive resources, and the way information is processed (Sweller, 1988, 2010; Paas and van Merriënboer, 2020).

*H3: Cognitive load mediates the relationship between AI-based educational technology use and critical thinking*.

However, students may differ in their ability to manage the cognitive demands associated with AI-supported learning. Self-regulated learning involves the active planning, monitoring, regulation, and evaluation of learning processes (Zimmerman, 2000; Pintrich, 2004). Students with stronger self-regulated learning abilities may be better equipped to manage cognitive demands, monitor their understanding, evaluate information, and adapt their learning strategies when necessary. In AI-supported learning environments, these regulatory processes may be particularly important because learners must determine how to use AI, evaluate AI-generated information, and integrate relevant information into their own knowledge structures (Kasneci et al., 2023). Consequently, self-regulated learning may weaken the negative association between cognitive load and critical thinking by helping learners regulate their cognitive engagement and maintain purposeful processing when confronted with demanding learning tasks.

*H4: Self-regulated learning moderates the relationship between cognitive load and critical thinking, such that the negative association between cognitive load and critical thinking is weaker among students with higher levels of self-regulated learning*.

Taken together, the proposed model suggests that the indirect relationship between AI-based educational technology use and critical thinking through cognitive load may vary according to students' self-regulated learning abilities. Specifically, the indirect effect is expected to differ according to the level of self-regulated learning because students with stronger self-regulated learning abilities may be better able to manage cognitive demands and sustain higher-order cognitive engagement. This proposition follows the logic of moderated mediation, in which cognitive load represents the mechanism through which AI-based educational technology use is associated with critical thinking, whereas self-regulated learning represents a condition that may alter the strength of the cognitive load–critical thinking relationship (Hayes, 2018). Accordingly, the indirect effect of AI-based educational technology use on critical thinking through cognitive load is expected to vary as a function of self-regulated learning.

*H5: Self-regulated learning moderates the indirect relationship between AI-based educational technology use and critical thinking through cognitive load, such that the indirect effect varies according to the level of self-regulated learning*.

Although there are many ways in which AI is being increasingly incorporated into educational technology used at post-secondary levels, most of the research that currently exists has primarily focused on learning efficiency, engagement, and academic performance; very little has been done to investigate the cognitive processes that underlie higher-order thinking (Kasneci et al., 2023; Zawacki-Richter et al., 2019). Furthermore, while there is some limited investigation of the relationship between cognitive load and self-regulated learning within AI-supported environments, few have examined these variables within an integrated model that examines both mediation and moderation. Understanding how learners engage with educational technology, including AI-based educational technology, will provide educators, developers, and researchers with a better sense of how to ensure that learners are using AI-based educational technology to enhance or undermine their critical thinking. Therefore, the present study addresses this limitation by proposing a moderated mediation model that examines the roles of cognitive load and self-regulated learning in the association between AI-based educational technologies and critical thinking. Specifically, the study aims to: (1) examine the relationships among AI-based educational technology use, cognitive load, self-regulated learning, and critical thinking; (2) investigate the mediating role of cognitive load in the relationship between AI-based educational technology use and critical thinking; and (3) determine whether self-regulated learning moderates the indirect relationship between AI-based educational technology use and critical thinking through cognitive load.

## 2 Methods

This section covers the research design, sample strategy, inclusion/exclusion criteria, data collection assessment measures, study procedure, ethical considerations, and statistical analysis used in testing study hypotheses.

### 2.1 Research design

This study employed a correlational research design and was conducted between December 2025 and February 2026. A correlational approach was considered appropriate because the study aimed to examine the relationships among AI-based educational technology use, cognitive load, self-regulated learning, and critical thinking among university students. The study protocol received ethical approval from Ethical Review Committee, Riphah Institute of Clinical and Professional Psychology, Riphah International University, Lahore, Pakistan (Reference: 067307; December, 8, 2025). All study procedures adhered to the ethical guidelines of the Helsinki Declaration and relevant guidelines for ethical research involving human subjects (World Medical Association, 2013). Participants were informed about the study's objectives, how responses would remain confidential, that the study was voluntary, and that they could withdraw at any time without consequence.

### 2.2 Study sample and sampling strategy

The study sample consisted of 480 undergraduate medical students recruited from government higher education institutions across Lahore, Punjab, Pakistan. All participants had prior experience using AI-based educational technologies, including generative AI platforms such as ChatGPT, AI-supported tutoring systems, and adaptive learning applications. There were 500 potential participants for data collection; however, after screening for incomplete responses, missing data and failing to meet all inclusion criteria, only 480 participant responses were used for final analysis. There were 290 (60.4%) females and 190 (39.6%) males, which indicates an overrepresentation of females within this study. The average participant age was 20.97 years (*SD* = 1.44), which reflects a relatively homogenous population of young adults. Nearly all participants (97%) reported that both of their parents were alive. All participants were undergraduate medical students enrolled in medical education programs. Regarding AI usage patterns, nearly all participants reported prior experience utilizing AI-based educational tools. The most utilized platform was ChatGPT. Most students reported using AI tools daily for approximately 1 h per day, primarily for concept development, academic support, medical learning, assignment assistance, and research-related activities. Participants were selected using convenience sampling to include individuals with medical backgrounds. The sample size was established based on an estimation of the sample size provided by the G^*^Power analysis in order to provide sufficient statistical power to detect a medium effect size in mediation and moderated mediation analyses.

### 2.4 Inclusion and exclusion criteria

To be eligible for participation, students were required to meet the following inclusion criteria: (1) be between 18 and 23 years of age, (2) be enrolled as full-time undergraduate students at an accredited university, and (3) have prior experience using AI-based educational technologies, such as ChatGPT or other AI-supported learning platforms, for at least one academic semester. Students were excluded if they had no prior experience with AI-based educational tools, were enrolled in non-accredited institutions, or were participating in vocational or technical training programs rather than undergraduate university education.

### 2.5 Assessment measures

#### 2.5.1 Artificial intelligence scale

The study used the AI Usage Scale, introduced by Vera (2023), to assess how often respondents used AI. The AI Usage Scale is a tool that evaluates beliefs, behaviors, and attitudes toward AI in the higher education setting and has 20 items arranged into four subdomains (such as perceived advantages, ethical concerns, institutional readiness, and expectations for the future. Each item was measured on a five-point Likert scale with responses of strongly disagree, somewhat disagree, neither agree nor disagree, somewhat agree, and strongly agree. The original scale had very good reliability (α = 0.89). In the current study, the scale also showed very good internal consistency (Cronbach's α = 0.88), thus providing evidence of reliable data collection.

#### 2.5.2 Critical thinking questionnaire

Critical thinking was assessed with the Critical Thinking Questionnaire (Kobylarek et al., 2022), which includes 25 items measuring the 6 components of Bloom's Taxonomy (remembering, understanding, applying, analyzing, evaluating, and creating). The questionnaire has both component scores and an overall critical thinking score. Internal consistency in this research was satisfactory (α = 0.76) as compared to the original Cronbach's alpha of α = 0.87.

#### 2.5.3 Cognitive load scale

Cognitive Load Scale, developed by Leppink et al. (2013), assessed germane, extraneous, and intrinsic cognitive load through 10 items on a 10-point Likert scale with ratings of “very low” to “very high.” Reliability coefficients have been reported in previous studies as being between α = 0.82 and 0.92. This study found that Cronbach's alpha for the scale was satisfactory at α = 0.69.

#### 2.5.4 Academic self-regulated learning questionnaire (ASLQ)

Self-regulated learning was assessed with the Academic Self-Regulated Learning Questionnaire (ASLQ), developed by Nambiar et al. (2022). The questionnaire includes 36 items that assess three stages of self-regulation: forethought, performance control, and self-reflection. The Cronbach alpha in the original study was very high (α = 0.90); likewise, in the current study, the reliability for the ASLQ was also very high (α = 0.88).

### 2.6 Ethical considerations

The study has followed the established guidelines for ethical conduct in research. Prior to participating in the study, all participants provided informed consent. Participants have been assured that they would remain anonymous and their information would be confidential. Participants were also advised of their right to discontinue their participation in the study at any time without being penalized. There is no anticipation of any potential harm (physical, psychological, or academic) to the participant. All collected data will be kept secure and will be used for research purposes only.

### 2.7 Procedure

Prior to data collection, ethical approval for the study was obtained from the Ethical Research Committee. Following this approval, formal permission was secured from the relevant institutional authorities to conduct the research. Authorization to use the selected assessment instruments was also obtained from the original authors of the scales. After all necessary approvals were granted, data collection was initiated. Participants completed a structured survey assessing four main domains: use of artificial intelligence technologies, perceived cognitive load, self-regulated learning behaviors, and critical thinking abilities. Before participating, students were informed about the objectives and purpose of the study and were assured that participation was entirely voluntary. They were also informed that all responses would remain anonymous and confidential and that they could withdraw from the study at any stage without any consequences. On average, completion of the questionnaire required approximately 15 to 20 min.

### 2.8 Data analysis

Data were analyzed using IBM SPSS Statistics (Version, 23). Pearson product–moment correlation coefficients were computed to examine the relationship among AI-based educational technology use, self-regulated learning, cognitive load, and critical thinking. Prior to conducting the main analyses, data screening procedures were performed to ensure the integrity of the dataset. Missing data were assessed, and cases with incomplete responses were excluded from the final analysis, resulting in a usable sample of 480 participants. Normality of the distribution was examined using the Kolmogorov–Smirnov test and inspection of skewness and kurtosis values, which confirmed that the data met the assumptions required for parametric analyses. Descriptive statistics, including means and standard deviations, were computed for all study variables to summarize their central tendencies and variability. All continuous variables were screened for outliers using z-score criteria (± 3.29), and no extreme outliers were identified. Common method bias was assessed using Harman's single-factor test; the results indicated that no single factor accounted for the majority of the variance, suggesting that common method variance was not a significant concern in this study.

To test the hypothesized mediation and moderated mediation effects, Hayes' PROCESS macro (Model 14) was employed. The independent variable (X) in this model is AI-based educational technology use; the mediator (M) is cognitive load; the dependent variable (Y) is critical thinking; and self-regulated learning (W) is a moderator of the path from cognitive load to critical thinking. Indirect and conditional effects were calculated using bias-corrected bootstrap confidence intervals based on 5,000 samples. Effects were considered statistically significant when the 95% bootstrap confidence interval did not include zero, consistent with Hayes‘ recommendations. For all other regression analyses, statistical significance was evaluated at *p* < 0.05.

## 3 Results

### 3.1 Descriptive statistics and correlation analysis

The descriptive statistics and Pearson Product-Moment Correlation Coefficients were calculated in order to determine how the use of AI-based educational technology relates to cognitive load, self-regulation of learning, and critical thinking. The descriptive statistics and correlation coefficients for the study variables are provided in Table 1.

**Table 1:** Descriptive statistics and correlations for study variables (*N* = 480).

Variables	*M*	*SD*	ATU	CT	CL	SRL
AI-based technology use (ATU)	83.22	14.51	–	0.46^**^	−0.09	0.48^**^
Critical thinking (CT)	91.08	11.57		–	−0.19^**^	0.62^**^
Cognitive load (CL)	46.65	15.88			–	−0.16^*^
Self-regulated learning (SRL)	132.35	20.60				–

*M*, mean; *SD*, standard deviation. ^*^p < 0.05; ^**^p < 0.01.

The use of AI in educational technology was found to be positively correlated with both critical thinking (*r* = 0.46, *p* < 0.01) and self-regulated learning (*r* = 0.48, *p* < 0.01). Use of AI-based educational technology, however, did show a statistically non-significant negative relationship with cognitive load (*r* = −0.09). Critical thinking was negatively correlated with cognitive load (*r* = −0.19, *p* < 0.01), while positively correlated with self-regulated learning (*r* = 0.62, *p* < 0.01). Cognitive load also was found to have a small but statistically significant (*r* = −0.16, *p* < 0.05) negative relationship with self-regulated learning. Therefore, the results of this study demonstrate that greater use of AI in education is positively associated with better critical thinking and self-regulated learning, whereas greater cognitive load is negatively associated with critical thinking.

### 3.2 Mediation analysis

Mediation analysis, through PROCESS Model 14, was conducted to assess if cognitive load mediated the association between AI-based educational technology use and critical thinking. The findings are presented in Table 2.

**Table 2:** Path coefficients for mediation of cognitive load.

Paths	Total effect		Direct effect		Indirect effect		95% CI	
	*B*	SE	*B*	SE	*B*	SE	LL	UL
AI → CT	0.17^*^	0.04	–	–	–	–	0.08	0.25
AI → CT	–	–	0.14^**^	0.04	–	–	0.06	0.22
AI → CL → CT	–	–	–	–	0.03^*^	0.01	0.01	0.05

AI, AI-based educational technology; CL, cognitive load; CT, critical thinking; SE, standard error; CI, 95% bias-corrected confidence interval; LL, lower limit; UL, upper limit. ^*^p < 0.05, ^**^p < 0.01.

The total effect of AI-based educational technology use on critical thinking was statistically significant [*B* = 0.17, SE = 0.04, 95% CI (0.08, 0.25)]. The direct effect of using AI-based educational technology on students' critical thinking skills remained statistically significant [*B* = 0.14, SE = 0.04, 95% CI (0.06, 0.22)], indicating that more frequent use of AI was associated with better critical thinking skills among students. Additionally, the indirect effect of AI use on critical thinking through cognitive load was found to be statistically significant [*B* = 0.03, SE = 0.01, 95% CI (0.01, 0.05)]. As the CI does not contain zero, this indicates that cognitive load partially mediates the relationship between the use of AI-based educational technology and critical thinking skills. Cognitive load decreased as the frequency of AI use increased, and this reduction in cognitive load was associated with improved critical thinking skills. Therefore, reduced cognitive load partially explains how increased use of AI-based educational technology is associated with improved critical thinking among students.

### 3.3 Moderated mediation analysis

**Figure 1:**
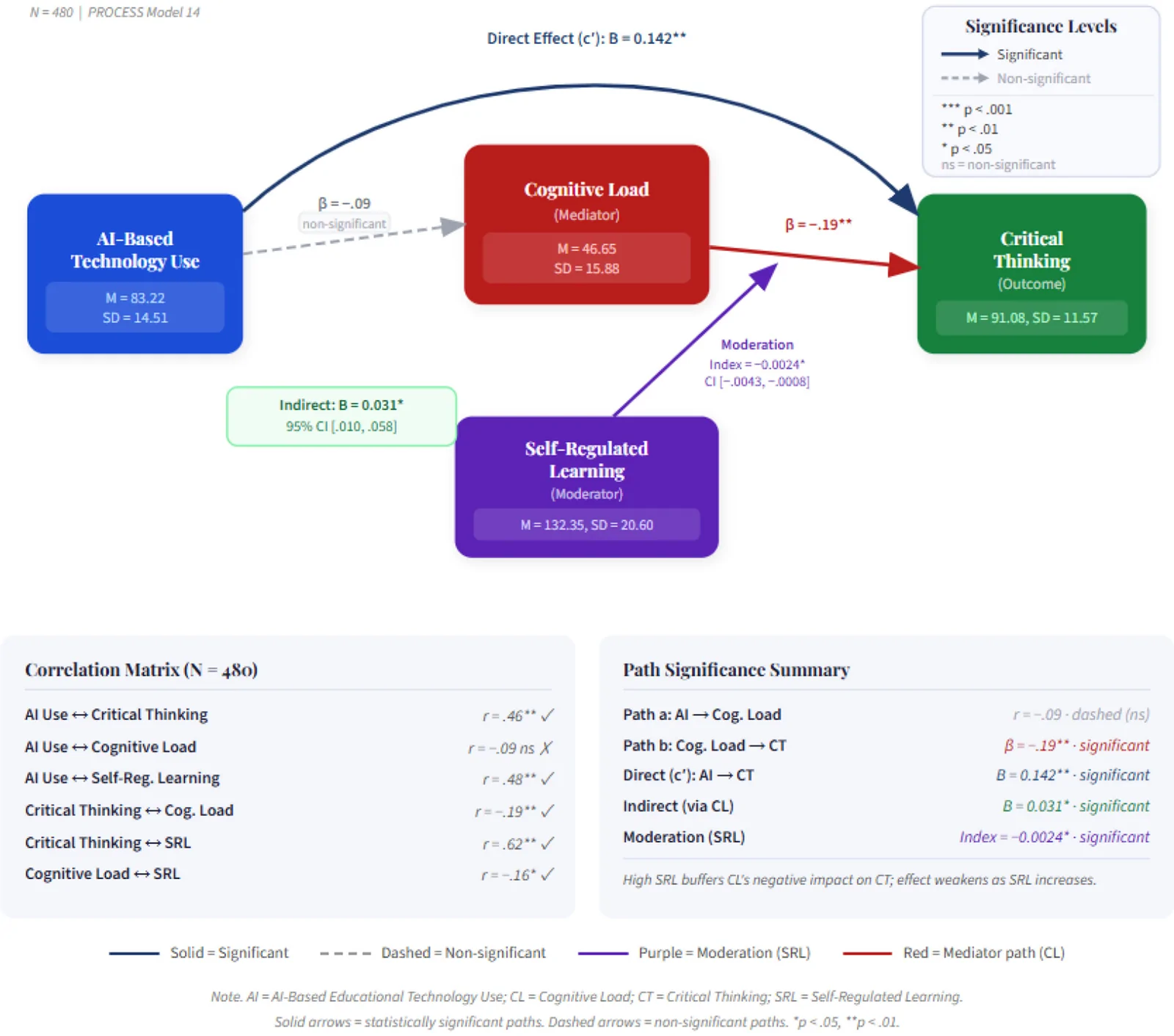
Conceptual model of the moderated mediation effect of self-regulated learning on the relationship between AI-based educational technology use, cognitive load, and critical thinking.

To examine whether self-regulated learning moderates the relationship between cognitive load and critical thinking, as well as the indirect effect of AI-based educational technology use on critical thinking via cognitive load, a moderated mediation analysis was conducted. The conceptual model is illustrated in Figure 1.

As shown in Table 3, self-regulated learning significantly moderated the relationship between cognitive load and critical thinking [*B* = 0.003, SE = 0.001, 95% CI (0.000, 0.007)], indicating that the negative association between cognitive load and critical thinking became weaker as self-regulated learning increased. Furthermore, the index of moderated mediation was statistically significant [*B* = −0.002, SE = 0.000, 95% CI (−0.004, −0.000)], as the confidence interval did not include zero. This finding indicates that the indirect effect of AI-based educational technology use on critical thinking through cognitive load significantly varied across levels of self-regulated learning. Specifically, at low levels of self-regulated learning, cognitive load exerted a significant negative effect on critical thinking. As levels of self-regulated learning increased, this negative effect gradually weakened, and at high levels of self-regulated learning, the impact of cognitive load on critical thinking became minimal or statistically non-significant. These results suggest that self-regulated learning functions as a protective or buffering factor, mitigating the detrimental influence of cognitive load on critical thinking. In other words, the mediating role of cognitive load is stronger among students with lower self-regulatory abilities and weaker among those with higher self-regulated learning skills.

**Table 3:** Path coefficients for moderated mediation of self-regulated learning.

Moderation and moderated mediation effects	*B*	SE	95% CI	
			LL	UL
CL × SRL	0.003^*^	0.001	0.000	0.007
Index of moderated mediation	−0.002^*^	0.000	−0.004	−0.000

SE, standard error; CI, confidence interval; LL, lower limit; UL, upper limit; CL, cognitive load; SRL, self–regulated learning. ^*^p < 0.05.

## 4 Discussion

The present study explored the associations among AI-based educational technology tools usage, cognitive load, self-regulated learning, and critical thinking among university students. The findings suggest that AI-based educational technology tool use is positively associated with students' critical thinking, supporting H1. The results are consistent with past literature on the possibility of the use of artificial intelligence to positively impact learning within higher education. For instance, Zawacki-Richter et al. (2019) stated that AI may support students in achieving a greater depth of understanding through applications such as adaptive learning systems or intelligent tutoring systems if they are properly integrated in instructional design and delivery. One possible explanation for this positive association is that AI tools can support higher-order cognitive functions by helping students organize information, clarify complex ideas, and structure their writing and reasoning processes. These functions may provide opportunities for students to analyze, evaluate, and improve their work, thereby supporting higher levels of cognitive complexity consistent with the revised taxonomy of educational objectives (Anderson et al., 2001). Thus, AI tools may function as assistive learning technologies that facilitate students' cognitive engagement with course material and support higher levels of intellectual involvement. Nevertheless, given the correlational design of the present study, the positive association between AI-based educational technology use and critical thinking should not be interpreted as evidence of a causal effect. Rather, the finding indicates a significant positive relationship between the two variables within the present sample.

Cognitive load was negatively associated with critical thinking, supporting H2. This finding is consistent with Cognitive Load Theory, which proposes that working memory has limited capacity and that excessive cognitive demands may restrict the cognitive resources available for meaningful learning and higher-order thinking (Sweller, 1988; Paas and van Merriënboer, 2020). When learners experience excessive cognitive demands, fewer cognitive resources may remain available for complex processes such as analysis, evaluation, reasoning, and critical judgment.

In the context of AI-supported learning, students may encounter both reduced and increased cognitive demands. AI-based educational technologies may reduce extraneous demands associated with searching for information, organizing content, and clarifying complex concepts. However, AI-generated information may also create additional demands because students must evaluate its accuracy, relevance, and credibility. The negative association observed between cognitive load and critical thinking suggests that greater cognitive demands may be associated with reduced capacity for higher-order cognitive engagement. This finding is consistent with the central assumptions of Cognitive Load Theory and previous research emphasizing the importance of managing cognitive demands to support meaningful learning (Sweller, 1988; Seufert et al., 2024).

The findings further indicated that cognitive load significantly mediated the relationship between AI-based educational technology tool usage and critical thinking, supporting H3. This finding suggests that cognitive load represents an important mechanism through which AI-based educational technology use is associated with critical thinking. AI-based educational technologies may influence the cognitive demands experienced by learners by helping them locate, organize, summarize, and interpret information. When such technologies reduce unnecessary cognitive demands, learners may have greater cognitive resources available for deeper reasoning and knowledge construction. Mayer (2009) similarly emphasized that learning environments that minimize unnecessary cognitive processing allow learners to devote greater cognitive resources to meaningful learning activities. Recent research has also suggested that appropriately designed AI-supported learning environments may help optimize cognitive processing during learning (Dong et al., 2025).

However, this mediation finding should be interpreted as an indirect statistical association rather than definitive evidence of a causal mechanism because the present study employed a cross-sectional design. Nevertheless, the finding provides empirical support for the proposed theoretical argument that cognitive load is an important cognitive mechanism linking AI-based educational technology use with critical thinking.

Self-regulated learning moderated the relationship between cognitive load and critical thinking, supporting H4. This finding is consistent with (Zimmerman, 2000) framework of self-regulated learning, which conceptualizes learning as a cyclical process involving planning, monitoring, and evaluating one's cognitive activities. Students with higher levels of self-regulated learning may be better equipped to regulate their cognition and adjust their learning strategies in response to cognitive challenges. In AI-supported learning environments, students with strong self-regulatory abilities may be more capable of determining how and when to use AI tools, monitoring their understanding, evaluating AI-generated information, and adapting their learning strategies. Consequently, self-regulated learning may help students manage cognitive demands and maintain meaningful cognitive engagement when learning tasks become complex.

Consistent with (Pintrich, 2004) emphasis on metacognitive monitoring and motivational regulation, the present findings extend the role of self-regulated learning to AI-mediated learning environments. The results suggest that self-regulated learning may function as an important learner characteristic that influences how cognitive load is associated with critical thinking.

Finally, the findings indicated that self-regulated learning moderated the indirect relationship between AI-based educational technology use and critical thinking through cognitive load, supporting H5. This finding indicates that the indirect relationship between AI-based educational technology use and critical thinking through cognitive load varied according to students' levels of self-regulated learning. Thus, the cognitive pathway linking AI-based educational technology use with critical thinking was not uniform across all learners.

Students with stronger self-regulated learning abilities may be better able to plan, monitor, and regulate their engagement with AI-supported learning activities. They may also be better equipped to evaluate AI-generated information and manage the cognitive demands associated with processing and integrating such information. Accordingly, self-regulated learning may shape the extent to which cognitive load is associated with critical thinking and, consequently, the strength of the indirect relationship between AI-based educational technology use and critical thinking.

Overall, the findings support the proposed moderated mediation model. Taken together, the results indicate that AI-based educational technology use is positively associated with critical thinking, whereas cognitive load is negatively associated with critical thinking and represents an important mediating mechanism in this relationship. In addition, self-regulated learning moderates both the relationship between cognitive load and critical thinking and the indirect relationship between AI-based educational technology use and critical thinking through cognitive load.

The findings therefore suggest that the educational value of AI-based technologies depends not only on the technological tools themselves but also on the cognitive demands associated with their use and students' ability to regulate their own learning. The potential for AI technologies to support higher-order thinking may consequently depend on the interaction between the cognitive characteristics of the learning environment and the self-regulatory capabilities of learners.

## 5 Limitations and future directions

The following limitations need to be taken into consideration. The first is the correlational design of this research, which limits researchers from drawing conclusions about causation. Future research should utilize either longitudinal or experimental designs to establish causal relationships. The second limitation is that the measurement of all of the variables used in this research utilized self-report assessment measures. They can cause response biases (e.g., social desirability) and/or inaccurate self-assessments. Future research could increase the validity of the findings of this research by utilizing objective indicators (e.g., behavioral logs of AI utilization, performance-based assessments of critical thinking, or physiological measures of cognitive load). The third limitation is that the sample consisted exclusively of undergraduate medical students. Therefore, caution should be exercised when generalizing the findings to students from other academic disciplines or the broader higher education population. Future research should include a more diverse and representative sample. A fourth limitation relates to the sampling strategy employed in this study. The use of convenient sampling, while practical, may have introduced selection bias, as participants who were more comfortable with or frequent users of AI tools may have been more likely to volunteer for the study. This limits the representativeness of the findings and caution should be exercised when generalizing the results to broader student populations. Future research should consider using stratified random sampling techniques to ensure more representative samples across diverse academic institutions. A final limitation relates to the geographical and institutional scope of the sample, as participants were recruited exclusively from government higher education institutions in Lahore, Punjab, Pakistan. The academic environment, institutional approaches toward AI integration, and demographic characteristics of students within this region may differ from those in other educational and cultural contexts. Consequently, the findings may not be fully generalizable to students from private institutions, other provinces, or different countries. Future research should replicate the study across diverse institutions and cross-cultural settings to enhance the external validity and broader applicability of the proposed moderated mediation model.

## 6 Implications

Beginning with theory, the current study extends Cognitive Load Theory (Sweller, 1988) and Self-Regulated Learning Models (Zimmerman, 2000; Pintrich, 2004) to explain how AI supports learners' higher-order thinking through an examination of the mediating effect of cognitive load and the moderating effect of self-regulated learning. Additionally, future studies could include additional learner attributes, such as digital self-efficacy, intrinsic motivation, and epistemological curiosity, to provide a greater understanding of how those learner characteristics affect the learning outcomes from AI-supported learning experiences in a variety of settings. The practical implications of this study indicate that the potential for AI to support learners' critical thinking is not unconditional. Specifically, AI technology appears to be most effective when students have the self-regulatory ability to utilize it strategically. Therefore, instructional designers who plan to integrate AI into their instruction should allow for some type of opportunity for students to engage in metacognition, reflect on their own learning, monitor their progress, and develop strategies to effectively use AI tools without developing cognitive overload. Future research should also investigate the long-term effects of AI tool usage on students' cognitive development, particularly examining whether sustained and strategic engagement with AI technologies over multiple academic semesters leads to durable improvements in critical thinking skills. Longitudinal research designs would be particularly valuable in this regard, as they would allow researchers to track developmental trajectories and identify critical periods during which self-regulated learning interventions may be most effective. Moreover, future studies should explore how different types of AI tools (e.g., generative AI vs. adaptive learning systems vs. AI-assisted feedback tools) may differentially impact cognitive load and critical thinking outcomes, enabling more nuanced and tool-specific recommendations for educators and curriculum designers. Cross-cultural and multi-institutional replication studies are also strongly encouraged to examine whether the moderated mediation model identified in this study holds across diverse higher education contexts and student populations with varying levels of technological access and AI familiarity.

In terms of policy making, the results of this study imply that colleges/universities need to implement a more balanced approach to integrating AI into their instruction. Instead of simply deciding whether or not to adopt AI technologies, institutions need to consider students' preparation to use AI tools, the instructional objectives of faculty members using AI tools and the overall support system available to students. Additionally, implementing AI literacy courses and instructional guidelines would assist in providing educators and policymakers with the information needed to ensure that AI is used in a manner that will have a positive impact on student learning. Finally, as new AI technologies are developed, future studies should investigate how new forms of AI technology will impact both the cognitive and metacognitive aspects of learning.

## 7 Conclusion

This study examined the relationship between university students‘ usage of AI-based instructional technology and critical thinking, using self-regulated learning as a moderator and cognitive load as a mediator. The results demonstrated that AI use was positively correlated with critical thinking and self-regulated learning, whereas cognitive load was negatively associated with both variables. Cognitive load partially mediated the relationship between AI use and critical thinking, indicating that AI tool may enhance higher order thinking by reducing unnecessary cognitive demands. Furthermore, self-regulated learning moderated this indirect relationship, such that the negative impact of cognitive load on critical thinking was weaker among students with higher self-regulatory abilities. Overall, the findings suggest that the effectiveness of AI-based educational technologies in promoting critical thinking depends not only on the tools themselves but also on students' ability to regulate their learning and manage cognitive demands. These results highlight the importance of integrating AI literacy and self-regulated learning strategies into higher education to maximize the educational benefits of AI-supported learning environments.

## Data Availability

The raw data supporting the conclusions of this article will be made available by the authors, without undue reservation.
